# Predicting the apolipoprotein E ε4 allele carrier status based on gray matter volumes and cognitive function

**DOI:** 10.1002/brb3.3381

**Published:** 2024-01-08

**Authors:** Hyug‐Gi Kim, Yunan Tian, Sue Min Jung, Soonchan Park, Hak Young Rhee, Chang‐Woo Ryu, Geon‐Ho Jahng

**Affiliations:** ^1^ Department of Radiology Kyung Hee University Hospital Seoul Republic of Korea; ^2^ Department of Medicine, Graduate School Kyung Hee University College of Medicine Seoul Republic of Korea; ^3^ Department of Biomedical Engineering, Undergraduate School, College of Electronics and Information Kyung Hee University Yongin‐si Gyeonggi‐do Republic of Korea; ^4^ Department of Radiology Kyung Hee University Hospital at Gangdong, Kyung Hee University College of Medicine Seoul Republic of Korea; ^5^ Department of Neurology Kyung Hee University Hospital at Gangdong, Kyung Hee University College of Medicine Seoul Republic of Korea

**Keywords:** aging, apolipoprotein E ε4 status, brain atrophy, cognitive decline, machine learning, prediction

## Abstract

**Background:**

Apolipoprotein E (ApoE) ε4 carriers have a higher risk of developing Alzheimer's disease (AD) and show brain atrophy and cognitive decline even before diagnosis.

**Objective:**

To predict ApoE ε4 status using gray matter volume (GMV) obtained from magnetic resonance imaging images and demographic data with machine learning (ML) methods.

**Methods:**

We recruited 74 participants (25 probable AD, 24 amnestic mild cognitive impairment, and 25 cognitively normal older people) with known ApoE genotype (22 ApoE ε4 carriers and 52 noncarriers) and scanned them with three‐dimensional (3D) T1‐weighted (T1W) and 3D double inversion recovery (DIR) sequences. We extracted GMV from regions of interest related to AD pathology and used them as features along with age and mini–mental state examination (MMSE) scores to train different ML models. We performed both receiver operating characteristic curve analysis and the prediction analysis of the ApoE ε4 carrier with different ML models.

**Results:**

The best model of ML analyses was a cubic support vector machine (SVM3) that used age, the MMSE score, and DIR GMVs at the amygdala, hippocampus, and precuneus as features (AUC = .88). This model outperformed models using T1W GMV or demographic data alone.

**Conclusion:**

Our results suggest that brain atrophy with DIR GMV and cognitive decline with aging can be useful biomarkers for predicting ApoE ε4 status and identifying individuals at risk of AD progression.

## Graphical abstract

The ApoE ε4 genotype might be carried by an elderly participant with a low MMSE score and GMV reduction in the amygdala and hippocampus. This result is important to identify individuals who have a high risk for AD progression in the future.

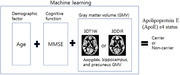

## 1 INTRODUCTION

Dementia is a major public health issue that affects millions of people worldwide. Alzheimer's disease (AD) is the most common form of dementia and is characterized by progressive cognitive impairment and brain atrophy (Breijyeh & Karaman, 2020; Molinder et al., 2021). Apolipoprotein E (ApoE) ε4 allele is the major genetic risk factor for AD and is associated with increased amyloid‐β (Aβ) deposition and accelerated brain atrophy (Corder et al., 1993; Suzuki et al., 2020). ApoE ε4 carriers have a higher risk of developing AD and show cognitive decline even before diagnosis (Corder et al., 1993). Therefore, it is important to identify ApoE ε4 carriers among older individuals who may have a high risk of AD progression.

Although the standard method to obtain ApoE genotype information is using blood samples, an alternative approach is to use brain imaging techniques to evaluate brain atrophy as a biomarker for ApoE ε4 status. Magnetic resonance imaging (MRI) is a widely used technique to measure gray matter volume (GMV) in regions‐of‐interest (ROI) related to AD pathology. However, different MRI sequences may have different sensitivities and specificities for detecting GMV changes. In this study, we investigated two MRI sequences: three‐dimensional (3D) T1‐weighted (T1W) and 3D double inversion recovery (DIR) (Jahng et al., 2016). Although 3D T1W is routinely used to evaluate GMV changes, the 3D DIR sequence has been shown to suppress both cerebrospinal fluid (CSF) and white matter signals more effectively than the T1W sequence, which may reduce the partial volume effect and improve the accuracy of GMV quantification (Jahng et al., 2016). Furthermore, based on this characterization, DIR could obtain whole‐brain‐high‐spatial resolution 3D images with clearly delineating cortical and deep gray matter structures (Pouwels et al., 2006). In addition, DIR could improve the signal‐to‐noise ratio and enhance lesion contrast to increase the description of lesions within the cerebral cortex as well as increase definition when evaluating mixed white matter‐gray matter lesions (Eichinger et al., 2017; Geurts et al., 2005).

Machine learning (ML) methods have been widely applied to neuroimaging data to classify or predict various neurological conditions. ML methods can combine multiple types of information, such as brain images and demographic data, to improve the accuracy and reliability of the predictions. By using a multiple‐kernel support vector machine (SVM), multiple types of information can be combined and compared with a single biomarker using single‐kernel learning. The prediction performance of this multiparametric approach may be higher than that of a single‐factor approach when classifying ApoE ε4 carriers from noncarriers. We developed a prediction method using an ML‐based optimized combination feature (OCF) set on GMV to classify and predict older cognitively normal (CN) participants, amnestic mild cognitive impairment (MCI), and mild and moderate AD patients (Kim et al., 2020). This OCF‐based method showed effective prediction performance for the MCI stage (Kim et al., 2020). This technique could be used to predict ApoE ε4 status with GMV and demographic parameters related to cognitive function. Few studies have evaluated the prediction of ApoE ε4 status with both GMV and demographic parameters such as age and the mini–mental state examination (MMSE) score (Honea et al., 2009; Kim et al., 2013; Yim et al., 2022). ApoE ε4 carriers are thought to show a close relationship between decreased cognitive function and brain atrophy, regardless of whether they are AD patients or not.

We hypothesized that the prediction of the ApoE ε4 carrier may be improved with DIR images due to the increase in sensitivity. Furthermore, combining MRI data and demographic data would improve the prediction of ApoE ε4 status compared to using either data source alone. To test our hypothesis, we recruited AD, amnestic MCI, and CN older participants with known ApoE genotypes and scanned them with both T1W and DIR sequences. In addition to MRI sequences, we also considered demographic data such as age and MMSE score as potential predictors of ApoE ε4 status. Age and MMSE score are known to be correlated with cognitive function and brain atrophy (Pini et al., 2016). We extracted GMV from ROIs related to AD pathology and used them as features along with age and MMSE score to train different ML models. We evaluated the models using receiver operating characteristic (ROC) curve analysis. The main objective of this study was to investigate the prediction of the ApoE ε4 status using GMV obtained from T1W or DIR images and/or demographic data with ML methods. The specific aims were as follows: (1) to evaluate the group classifications between ApoE ε4 carriers and noncarriers using ROC curve analysis with GMV obtained from T1W or DIR images and/or demographic data; (2) to compare the performance of different ML models for predicting ApoE ε4 status using different combinations of features.

## 2 MATERIALS AND METHODS

### 2.1 Participants

This study was approved by our institutional review board, and informed consent was obtained from all participants. Participants were prospectively recruited in the Brain Neurological Center of our institution. All participants were Korean. A detailed medical history was provided by them, and they underwent a neurologic examination, standard neuropsychological testing, genotype evaluation, and MRI scan. A neuroradiologist with 12 years of imaging experience evaluated the brain MR images for each participant to determine any evidence of prior cortical infarctions or other space‐occupying lesions.

A standardized neuropsychological battery in Korea that covers the five cognitive subsets: attention, memory, language, visuospatial function, and frontal/executive function was used to assess cognitive function (Ahn et al., 2010). This test is included in the Korean version of the MMSE for global cognitive ability. Probable AD patients with mild and moderate dementia were recruited based on the criteria of the National Institute of Neurological and Communicative Disorders and Stroke‐Alzheimer Disease and Related Disorders Association (Dubois et al., 2007; McKhann et al., 1984). They were defined as those with clinical dementia rating scores of 0.5, 1, or 2 based on the results of the neuropsychological examination. In addition, amnestic MCI subjects were included in the study based on the Petersen criteria (Petersen, 2016; Petersen et al., 1999, 2001). Finally, CN older participants were selected from healthy volunteers who did not have a medical history of neurological disease, who showed normal results on detailed evaluation according to the Korean normative database, and who did not have abnormal brain tumors or strokes by MRI.

Blood samples were taken from participants to determine ApoE ε genotypes using a restriction enzyme polymerase chain reaction technique (GC Corp, Yougin‐si, Korea). Participants were then divided into two groups depending on the presence or absence of the ApoE ε4 allele: ε2/ε3, ε3/ε3, ε2/ε4, and ε3/ε4. Carriers and noncarriers of this risk factor for AD were identified by this division.

A total of 77 participants were included in this study. Three participants did not provide blood samples, although they had undergone SNSB examinations and MRI scans. Therefore, the 74 participants were differentially diagnosed as 25 probable AD, 24 amnestic MCI, and 25 CN. In the noncarrier group, there were 12 AD (23.1%), 19 MCI (36.5%), and 21 CN (40.4%) participants. In the carrier group, there were 13 AD (59.1%), 5 MCI (22.7%), and 4 CN (18.2%) participants. Of the 74 participants, 52 were ApoE ε4 allele noncarriers (70.27%) but 22 were carriers (29.73%). ApoE ε3/ε3 homozygotes were the dominant genotype in the noncarrier group (45/52, 86.54%), whereas ApoE ε3/ε4 was the main genotype among carriers (21/22, 95.45%). There were no participants with the ε4/ε4 allele. In both the MCI and CN groups, most of the participants were ApoE ε4 noncarriers. Table 1 summarizes the genotypes and demographic characteristics of participants in the study, as well as the MMSE score.

**TABLE 1:** Demographic characteristics and the apolipoprotein E (ApoE) genotypes of study participants.

Group		Noncarrier	Carrier	Sum or *p*‐value
Subjects		52 (70.27%)	22 (29.73%)	74
AD		12 (23.1%)	13 (59.1%)	25
MCI		19 (36.5%)	5 (22.7%)	24
CN		21 (40.4%)	4 (18.2%)	25
Genotypes	ε2/ε3:	7 (13.46%)	0	7
	ε2/ε4	0	1 (4.55%)	1
	ε3/ε3	45 (86.54%)	0	45
	ε3/ε4	0	21 (95.45%)	21
Age^a^ (years)		66.4 ± 8.8	70.7 ± 5.7	*.042*
Sex^b^ (Male/Female)		18/34	5/17	*χ* ^2^ = .026
				*p* = .872
MMSE^a^		24.8 ± 5.6	19.9 ± 6.4	*.001*

Abbreviations: AD, Alzheimer's disease; CN, cognitively normal; MCI, mild cognitive impairment; MMSE, mini–mental state examination.

^a^ Age and MMSE scores are presented as means ± standard deviation (SD). The *p*‐values show the result of the two‐sample *t*‐test of each parameter between ApoE ε4 allele carriers and non‐carriers.

^b^ Sex was compared by the chi‐square test.

### 2.2 MRI acquisition

MR images were obtained with a 3 Tesla MRI scanner (Achieva, Philips Medical System) with an 8‐channel head coil. Both 3D T1W and 3D DIR sequences were scanned for each participant to determine their GMVs. The isotropic sagittal structural 3D T1W images (T1WIs) were acquired from the turbo‐field echo sequence with the following imaging parameters: repetition time (TR)/echo time (TE)/inversion time (TI) = 8.1/3.7/1013 ms, flip angle = 8°, matrix = 236 × 236, and voxel size = 1 × 1 × 1 mm^3^. The scan time for the 3D T1W sequence was 4 min and 48 s.

The following parameters were used to acquire the 3D DIR images in this study. The first TI of 2930 ms was used to suppress CSF signals, and the second TI of 350 ms was used to suppress white matter signals (Jahng et al., 2016). After applying two inversion pulses, signals were acquired by turbo spin‐echo (TSE) imaging (Jahng et al., 2016) with the following imaging parameters: TR/effective TE = 8000/100 ms, echo‐spacing = 10.2 ms, echo‐train length = 11, overcontiguous slice thickness = 4 mm, reconstructed slice thickness = 2 mm, number of slices = 50, acquisition voxel size = 0.9 × 1.12 × 4, reconstructed voxel size = 0.45 × 0.45 × 2, TSE factor = 43, sensitivity‐encoding factor = 2.5, and number of averages = 1. The scan time for the 3D DIR sequence was 6 min and 16 s. In addition, T2‐weighted images and fluid‐attenuated inversion recovery images were also acquired to identify any brain abnormalities.

### 2.3 Preprocessing of images

The following preprocessing analysis was performed using Statistical Parametric Mapping‐version 12 (SPM12) software (Wellcome Department of Imaging Neuroscience, University College, London, UK). First, brain tissue volumes were obtained by segmenting both 3D DIR and 3D T1WIs. Second, study‐specific templates for 3D T1W and 3D DIR were created separately using segmented brain tissue volume maps and the “Diffeomorphic Anatomical Registration Through Exponentiated Lie Algebra (DARTEL)” algorithm (Ashburner, 2007). Third, the segmented GMV maps of both 3D DIR and 3D T1WIs were applied to the study‐specific DIR and T1W templates, respectively. Finally, the spatially normalized GMV maps for both DIR and T1W were smoothed using a Gaussian kernel of 8 × 8 × 8 mm full width at half maximum to perform the following voxel‐based statistical analyses.

### 2.4 Statistical analyses

#### 2.4.1 Demographic data and clinical outcome scores

Age and MMSE scores were compared between ApoE ε4 carrier and noncarrier groups using the two‐sample *t*‐test. The chi‐squared test was used to compare sex.

#### 2.4.2 Voxel‐based analysis of GMV map

A voxel‐based two‐sample *t*‐test was applied with the participant's age as a covariate to compare GMVs between the ApoE ε4 carrier and noncarrier groups for each sequence. The significance level was set at *α* = .05 with correcting multiple comparisons using the false discovery rate method and with the minimum cluster size with at least 100 contiguous voxels. This study was mainly performed to define the specific brain areas for the ROI analysis.

#### 2.4.3 ROI‐based analysis of GMV value

GMV values for each participant and each sequence were obtained by defining ROIs using the following two different methods: First, ROIs were defined at the disease‐specific brain areas such as the amygdala, hippocampus, and precuneus. Second, additional ROIs were defined in the areas of significant differences in GMV between the two groups on the voxel‐based analysis, such as the posterior cingulate, middle frontal gyrus (MFG), and middle temporal gyrus (MTG). These areas were automatically traced on the brain atlas space using the WFU_PickAtlas software toolbox (fmri.wfubmc.edu/software/pickatlas). The mean value of GMV for each participant and each sequence was obtained from the six ROIs using the Marsbar software (Matthew Brett, http://marsbar.sourceforge.net).

The following three analyses were performed using ROI data. First, a two‐sample *t*‐test was performed to compare GMV between ApoE ε4 carrier and noncarrier groups for each ROI area and each sequence. Second, a ROC curve analysis was performed for each sequence to evaluate the differentiation between ApoE ε4 carriers and noncarriers using the demographic data of age, gender, or MMSE scores and/or GMV for each ROI area. Furthermore, ROC curves between T1W and DIR were compared for each ROI. For those ROI analyses, *α* < .05 was used to determine the significance level. Third, a logistic regression analysis was performed to evaluate the association between ApoE4 status and MMSE scores or GMVs from the T1WI or DIR technique for each ROI. The statistical analysis was performed using the MedCalc statistical software (http://www.medcalc.org/). Finally, ML methods were used to predict ApoE ε4 carriers and noncarriers using the following two‐step analyses. In the first step, the features were selected using the OCF setting method to minimize the curse of dimensionality from nine parameters of age, sex, MMSE, and GMV values from six ROIs for each sequence (Kim et al., 2020). The number of combinations was estimated to be a combination of features from a minimum of single to a maximum of nine from all features. The total combination set for the combination‐based feature selection was calculated as 511 based on the equation *nC_r_* (*n* = 9 and 1 ≤ *r* ≤ 9), where *n* represents the number of features, and *r* represents the number of features being chosen at a time. In the next step, three different SVM kernels which are the linear kernel (first‐order polynomial SVS or SVM1), quadratic kernel (second‐order polynomial SVM or SVM2), cubic kernel (third‐order polynomial SVM or SVM3) as well as the two additional models of Bootstrap‐Aggregated decision tree or Tree Bagger (TB) (Meinshausen, 2006) and kernel‐based naive Bayes (NB) (Manning et al., 2008) models were used to train the SVM kernel classifiers to predict the ApoE ε4 carriers and noncarriers groups using each OCF set. The dataset was randomly evaluated for the test dataset using a threefold cross‐validation technique. The result of this analysis was presented by the area under curve (AUC) calculations for each OCF set and with each kernel type from threefold cross‐validation sets. The ratio between the training set and the test set was 7‐to‐3. Furthermore, we performed the three‐group classification with the ML models of first‐, second‐, and third‐order SVM, TB, or NB using features of age, sex, MMSE scores, ApoE state, and all ROIs’ GMV for each sequence. In this analysis, we also used the threefold cross‐validation sets.

## 3 RESULTS

### 3.1 Demographic data

The carrier group was significantly older than the noncarrier group (*p* = .042) (Table 1). The MMSE scores were significantly lower in the carrier group than in the noncarrier group (*p* = .001). No significant difference was found between the two groups in sex (*χ* ^2^ = .026, *p* = .872). More female than male participants were observed for both groups.

### 3.2 Comparisons of GMVs between the carrier and noncarrier groups

#### 3.2.1 Voxel‐based comparison

**FIGURE 1:**
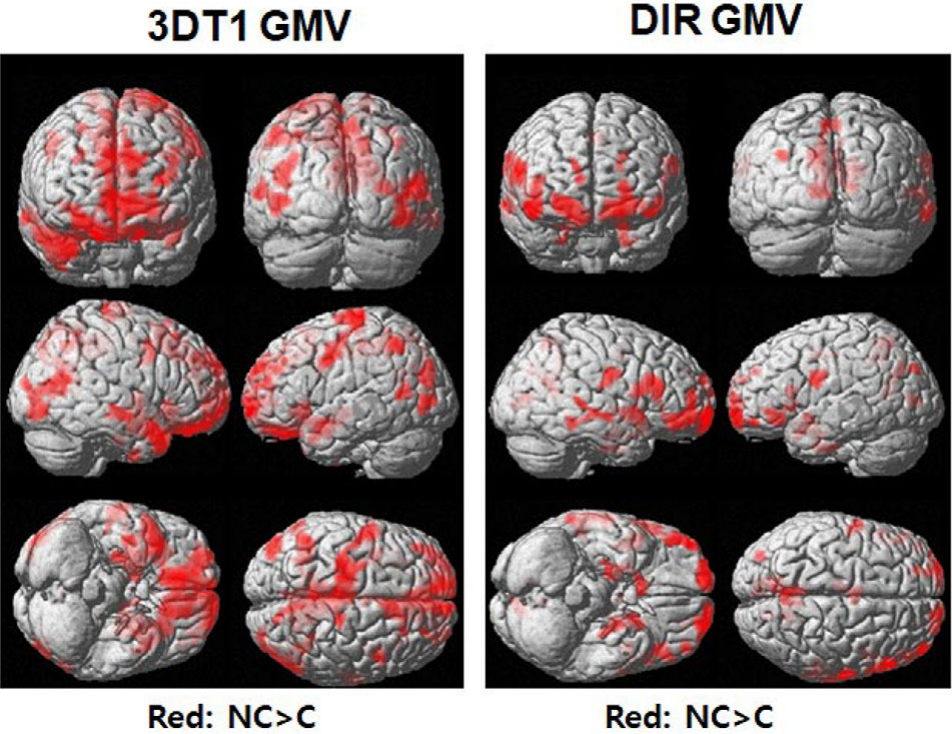
Results of the voxel‐based two‐sample *t*‐test analyses of gray matter volume (GMV) between the two groups of apolipoprotein E (ApoE) ε4 carrier and noncarrier for three‐dimensional T1 (3DT1) and double inversion recovery (DIR) sequence.

Significant GMV reductions in both DIR and T1WIs were observed in the ApoE ε4 carrier group compared with the noncarrier group (Figure 1). The areas of the GMV reduction in both sequences were precuneus, frontal gyrus, and temporal gyrus (Table S1). Compared to T1W, the areas of GMV loss in the DIR were extended to the anterior cingulate, posterior cingulate, and parahippocampal gyrus.

#### 3.2.2 ROI‐based comparison

**FIGURE 2:**
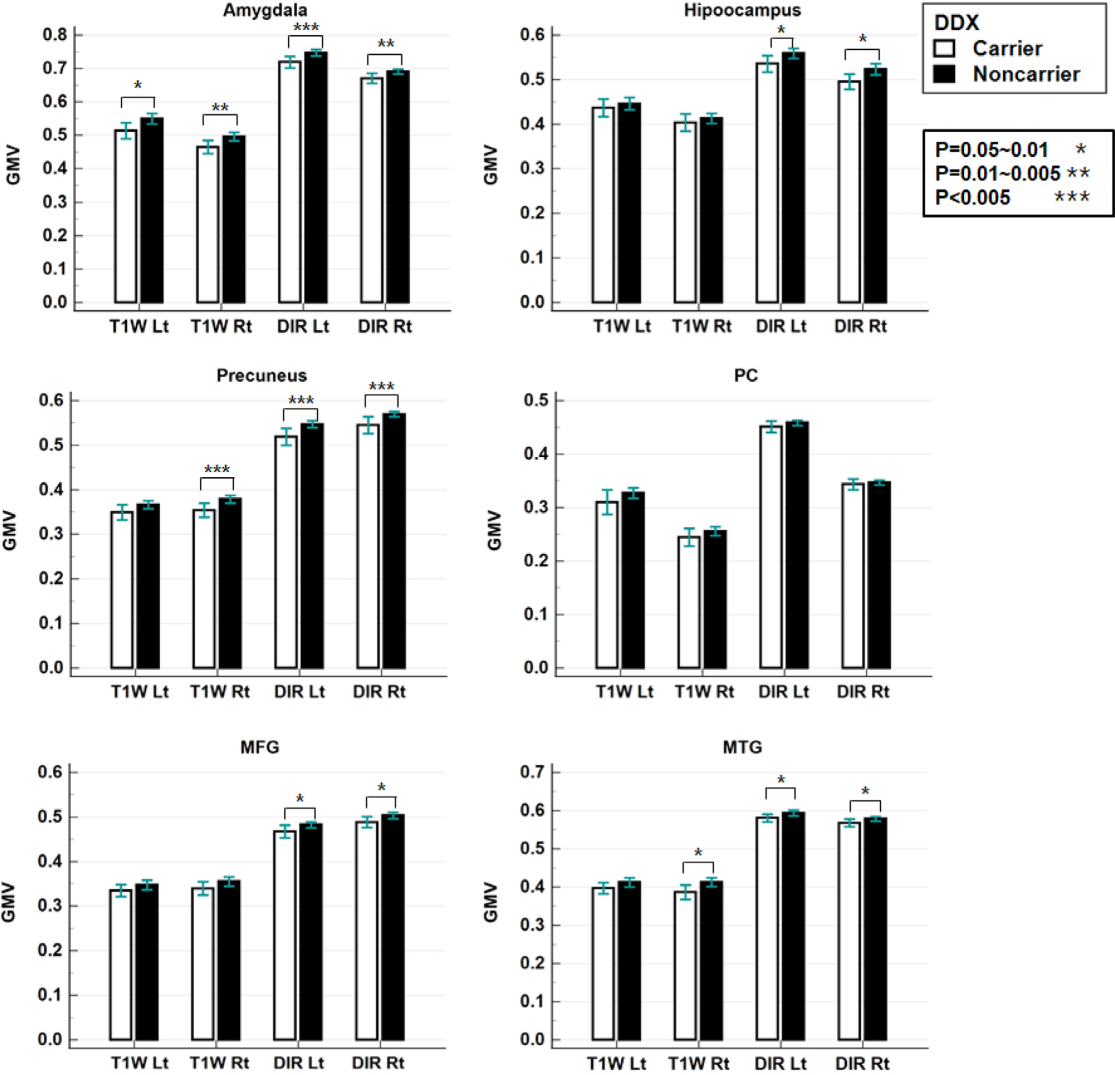
Results of the two‐sample *t*‐test analyses of the gray matter volume (GMV) value between the two groups of apolipoprotein E (ApoE) ε4 carrier and noncarrier for three‐dimensional T1‐weighted (T1W) and double inversion recovery (DIR) sequence at each brain area. Brain region‐of‐interest (ROI) areas were the amygdala, hippocampus, precuneus, posterior cingulate, middle frontal gyrus (MFG), and the middle temporal gyrus (MTG) at the right (Rt) or left (Lt) side.

First, GMV in each ROI was lower in the carrier group than in the noncarrier group in all ROI areas (Figure 2, Table S2). The carrier group had significantly lower GMV than the noncarrier group in the bilateral amygdala, right precuneus, and right MTG for both T1W and DIR. Only the DIR image showed additional areas of significant GMV reduction in the carrier group in the bilateral hippocampus, the left precuneus, the bilateral MFG, and the left MTG. Second, the results of the logistic regression analysis between ApoE state and MMSE scores or GMVs from T1WI or DIR sequence were listed in Table 4. Increased MMSE score was associated with a reduced odds ratio (OR) of the ApoE4‐carrier (OR = .876, *p* = .003). With T1WIs, more GMV loss was associated with increased OR of ApoE4‐carrier in left amygdala (OR = .889, *p* = .020), right amygdala (OR = .849, *p* = .013), right precuneus (OR = .788, *p* = .007), and MFG (OR = .852, *p* = .020). With DIR images, more GMV loss was associated with increased OR of ApoE4‐carrier in the left amygdala (OR = .807, *p* = .006), right amygdala (OR = .776, *p* = .011), left hippocampus (OR = .872, *p* = .031), right hippocampus (OR = .864, *p* = .020), left precuneus (OR = .756, *p* = .007), right precuneus (OR = .738, *p* = .010), left MFG (OR = .805, *p* = .031), and right MFG (OR = .789, *p* = .030). Therefore, hippocampus and precuneus areas were more associated with DIR than T1W.

### 3.3 Prediction of apolipoprotein E ε4 allele carriers

#### 3.3.1 ROC analysis

Table 2 shows the result of ROC curve analysis. The ApoE ε4 carrier group was significantly differentiated from the noncarrier group by age (AUC = .670, *p* = .007), MMSE score (AUC = .730, *p* < .001), and the combination of both age and MMSE score (AUC = .758, *p* < .0001). With T1WI, GMV at the left (AUC = .67, *p* = .011) and right (AUC = .70, *p* = .004) amygdala, right precuneus (AUC = .690, *p* = .006), and right MTG (AUC = .650, *p* = .032) significantly differentiated the carrier group from the noncarrier group. With the DIR image, GMV in all areas except the posterior cingulate significantly differentiated the carrier group from the noncarrier group. The AUC value was improved by combining GMV and the demographic data of both age and MMSE scores in each sequence, but it was similar to that of the combination of both age and MMSE scores. No significant difference was found between DIR GMV and T1W GMV for all ROIs in AUC values.

**TABLE 2:** Results of receiver operating characteristic (ROC) curve analyses with the demographic data or gray matter volume (GMV) for the group classifications between apolipoprotein E (ApoE) ε4 carrier and noncarrier.

Demographic data^a^				
Parameters	Sensitivity (SE)	Specificity (SP)	AUC	*p*
Age	68.18	71.15	.670	.007
Gender	77.27	34.62	.560	.293
MMSE	68.18	80.77	.730	<.001
Age + MMSE	77.27	73.08	.758	<.0001

**GMV**										
ROI	Side	**T1W GMV**				**DIR GMV**				ROC
		SE	SP	AUC	*p*	SE	SP	AUC	*p*	*p* ^b^
Amygdala (Amyg)	Lt	63.64	65.38	.670	.011	77.27	59.62	.700	.004	.671
	Rt	68.18	69.23	.700	.004	63.64	76.92	.700	.005	.979
Hippocampus (Hippo)	Lt	90.91	28.85	.540	.594	90.91	44.23	.660	.012	.266
	Rt	95.45	25.00	.590	.242	81.82	63.46	.710	.002	.200
Precuneus	Lt	40.91	86.54	.620	.115	68.18	67.31	.700	.002	.406
	Rt	95.45	42.31	.690	.006	72.73	57.69	.690	.004	.967
PC	Lt	50.00	78.85	.590	.290	50.00	82.69	.630	.095	.671
	Rt	36.36	86.54	.600	.209	63.64	65.38	.600	.195	1.000
MFG	Lt	95.45	36.54	.600	.153	90.91	42.31	.640	.050	.688
	Rt	81.82	48.08	.620	.078	86.36	48.08	.660	.021	.703
MTG	Lt	90.91	40.38	.620	.078	86.36	51.92	.700	.002	.308
	Rt	68.18	63.46	.650	.032	63.64	76.92	.680	.015	.782
Age + MMSE + Amyg	Rt	72.73	78.85	.747	.0002	68.18	82.69	.759	.0001	.744
Age + MMSE + Hippo	Rt	77.27	76.92	.761	<.0001	72.73	75.00	.747	.0001	.493
Age + MMSE + MTG	Rt	77.27	75.00	.757	<.0001	77.27	71.15	.760	<.0001	.668

*Note*: Age + MMSE + Amyg: Age + MMSE + Rt amygdala; Age + MMSE + Hippo: Age + MMSE + Rt hippocampus; Age + MMSE + MTG: Age + MMSE + Lt MTG.

Abbreviations: AUC, area under the ROC curve; DIR, double inversion recovery; Lt, left; MFG, middle frontal gyrus; MMSE, mini–mental state examination; MTG, middle temporal gyrus; PC, posterior cingulate; Rt, right; T1W, T1‐weighted.

^a^ The demographic data of age, sex, and MMSE score and GMV at the region‐of‐interest (ROI) areas for each sequence were used for the receiver operating characteristic (ROC) analyses.

^b^ Comparison of ROC curves between T1W and DIR.

#### 3.3.2 Prediction analysis with machine learning

Table 3 shows the prediction result using ML analysis. AUC values with DIR GMVs were higher than those with T1W GMVs. The highest AUC value was obtained with the cubic SVM (SVM3) model by combining the five features of age, MMSE score, and DIR GMVs at the amygdala, hippocampus, and precuneus (AUC = .88). The second highest AUC value was obtained with the quadratic SVM (SVM2) model by combining the four features of age, MMSE score, and DIR GMVs at the hippocampus and MTG (AUC = .84). Without using any demographic data of age, MMSE score, and sex, the DIR GMVs combining both amygdala and precuneus provided AUC = .78 with the linear SVS (SVM1) model. With T1W GMVs, the AUC values were .81 for the TB model by combining the three features of sex, age, and T1W GMVs at the amygdala and .81 for the NB model by combining the four features of age, MMSE score, and T1W GMVs at the amygdala, and hippocampus.

**TABLE 3:** Prediction analysis results using the machine learning models with the demographic data and the gray matter volume (GMV) from the two different magnetic resonance imaging (MRI) sequences to differentiate between apolipoprotein E (ApoE) ε4 carriers and noncarrier groups.

	T1W GMV					DIR GMV				
Model	SVM1	SVM2	SVM3	TB	NB	SVM1	SVM2	SVM3	TB	NB
AUC	.76	.79	.76	.81	.81	.78	.84	.88	.80	.81
Num	234	175	224	416	240	40	209	248	308	443
Sex	0	0	0	1	0	0	0	0	1	1
Age	1	1	1	1	1	0	1	1	0	1
MMSE	1	0	1	0	1	0	1	1	0	0
Amygdala	1	1	1	1	1	1	0	1	1	1
Hippocampus	0	0	0	0	1	0	1	1	1	1
Precuneus	1	1	0	0	0	1	0	1	0	1
PC	0	1	0	0	0	0	0	0	1	0
MFG	1	1	0	0	0	0	0	0	0	1
MTG	0	1	0	0	0	0	1	0	0	1
Feature *N*	5	6	3	3	4	2	4	5	4	7

*Note*: Prediction models: SVM1, the first‐order SVM or linear SVM model; SVM2, the second‐order polynomial SVM model or the quadratic SVM; SVM3, the third‐order polynomial SVM or cubic SVM or random forest model; TB, the Bootstrap‐Aggregated decision tree or Tree Bagger model; NB, the kernel‐based naive Bayes or kernel‐naive Bayes model. Num is the number of the total combined feature selection. One (1) indicates the selected parameter to calculate AUC, but zero (0) does the unselected parameter.

Abbreviations: AUC, area under the ROC curve; DIR, double inversion recovery; MFG, middle frontal gyrus; MMSE, mini–mental state examination; MTG, middle temporal gyrus; PC, post cingulate; SVM, support vector machine; T1W, T1‐weighted.

#### 3.3.3 Three‐group classification using ML models for each sequence

Table 5 lists the results of the three‐group classification with the different ML models using both demographic data and GMV values obtained from six different brain areas for each imaging sequence. The largest AUC values were .763 for T1W GMV with the first‐order SVM model and .830 for DIR GMV with the third‐order SVM model. The largest AUC values listed in this table for each sequence were always obtained using 10 features of the combination of age, sex, MMSE scores, ApoE state, and 6 ROIs’ GMV. Therefore, we used all 10 features for both sequences. Because SVM3 shows the largest AUC value, we show the confusion matrix of SVM3 for both T1W GMV and DIR GMV in Supplementary Figure S1 to show the model performance. For T1W GMV, the correct predictions were 60% (15/25) for CN, 45.8% (11/24) for MCI, and 72% (18/25). One CN participant was predicted as AD and two AD participants were predicted as CN. For DIR GMV, the correct predictions were 68% (17/25) for CN, 83.3% (20/24) for MCI, and 80% (20/25). All CN participants were predicted as CN or MCI and three AD participants were predicted as CN.

## 4 DISCUSSION

### 4.1 GMV reduction in APOE ε4 carriers

Both DIR and T1W techniques determined that GMV was significantly reduced in ApoE ε4 carriers relative to noncarriers in several brain areas (Figures 1 and 2) which was a consistent finding in previous reports (Bailey et al., 2015; Coughlan et al., 2021; Hashimoto et al., 2001; Liu et al., 2010). The carrier group showed GMV reduction by DIR in the bilateral hippocampus, bilateral MFG, and bilateral MTG, but was not by gradient‐echo T1W, indicating that DIR was more sensitive than T1W in detecting GMV loss. The 3D T1W TFE sequence, which is similar to the MPRAGE technique, is a gradient‐echo sequence that is usually affected by inhomogeneity in an applied magnetic field that induces susceptibility artifacts. However, DIR is a spin‐echo sequence, which is less affected by field inhomogeneity artifacts. Gray matter segmentation may also be affected by B0 field inhomogeneity in the frontal and temporal areas. Therefore, gradient‐echo T1W may underestimate GMV in the temporal area but overestimate in the frontal area of the brain (Diaz‐de‐Grenu et al., 2011). Furthermore, white matter signals are reduced in DIR images by suppressing both CSF and white matter. ApoE ε4 state showed a negative association with MMSE scores (Table 4). Furthermore, ApoE ε4 was negatively associated with GMV with both T1WI and DIR. DIR GMV was statistically significant in more ROIs compared with T1WI GMV, which indicates that the DIR sequence was more sensitive. Therefore, DIR may have an advantage over T1W in evaluating the characteristics of the ApoEε4 carriers by evaluating GMV loss in the brain.

**TABLE 4:** Results of logistic regression analysis between apolipoprotein E (ApoE) state and the mini–mental state examination (MMSE) scores or gray matter volume (GMV) values for each three‐dimensional (3D) T1‐weighted image (T1WI) or double inversion recovery (DIR) sequence in each region‐of‐interest (ROI).

Demographic data			
Parameters	OR	95% CI	*p*‐Value
MMSE scores	.876	.802–.957	.003

**GMV**							
ROIs	Side	**T1WI**			**DIR**		
		OR	95% CI	*p*‐Value	OR	95% CI	*p*‐Value
Amygdala	Lt	.889	.805–0.981	.020	.807	.693–.939	.006
	Rt	.849	.745–0.966	.013	.776	.639–.943	.011
Hippocampus	Lt	.959	.861–1.068	.443	.872	.769–.988	.031
	Rt	.944	.832–1.072	.374	.864	.764–.977	.020
Precuneus	Lt	.864	.741–1.006	.060	.756	.617–.927	.007
	Rt	.788	.662–0.937	.007	.738	.587–.929	.010
PC	Lt	.903	.797–1.023	.110	.826	.640–1.066	.142
	Rt	.894	.761–1.052	.178	.912	.698–1.192	.499
MFG	Lt	.904	.793–1.030	.130	.805	.661–.980	.031
	Rt	.852	.744–0.975	.020	.789	.637–.977	.030
MTG	Lt	.905	.786–1.043	.167	.827	.681–1.004	.054
	Rt	.884	.764–1.022	.097	.796	.629–1.007	.057

*Note*: The Korean version of the mini–mental state examination (MMSE) scores and gray matter volume (GMV) values at the region‐of‐interest (ROI) areas for each sequence were used for the logistic regression analysis with apolipoprotein E (ApoE) state. In this table, we list the result of odds ratio (OR) and 95% confidence interval (CI) with *p*‐value for each analysis.

Abbreviations: Lt, left; MFG, middle frontal gyrus; MTG, middle temporal gyrus; PC, posterior cingulate; Rt, right.

### 4.2 Prediction of apolipoprotein E ε4 allele carriers

The ML method showed that the highest AUC value was obtained when the following four features were included in the prediction analysis with the cubic kernel SVM (SVM3) model: DIR GMVs in the amygdala, hippocampus, and precuneus as well as demographic values of ages and MMSE scores (AUC = .88) (Table 3). Larger AUC values were obtained with DIR GMVs than T1W GMVs. High AUC values were obtained when age and MMSE scores were included as features. High AUC values were also obtained when GMVs in both the amygdala and hippocampus were included as features. With T1W GMV, higher AUC values were shown by the ML methods of TB and NB than any kernels of SVM models. Without using any demographic values including MMSE scores, a high AUC value (AUC = .78) was shown by DIR GMVs in the amygdala and precuneus with the linear SVM model.

ApoE is a glycoprotein involved in cholesterol homeostasis and lipid metabolism, is produced mainly by hepatocytes and astrocytes, and is found in plasma and CSF. The ApoE e4 allele is one of the most notorious common genetic risk factors, with the potential to increase AD risk up to 15‐fold when homozygous, and further adverse effects on lipid profiles and cardiovascular diseases (Lumsden et al., 2020). The human ApoE gene has two genetic polymorphisms, ε2 and ε3, which inhibit Aβ protein aggregation, but ApoE ε4, which is called a carrier, is less effective than ApoE ε3, which is called a noncarrier. Therefore, more and earlier amyloid deposits are found in AD patients with ε4 than those without ε4 (Nicoll et al., 2011; Vemuri et al., 2010). A previous study showed that the risk of AD increased almost four times as the number of ApoE ε4 alleles in families with late‐onset AD increased, whereas the mean age at onset decreased from 84 to 68 years (Corder et al., 1993; Verghese et al., 2011). In particular, a more than 10‐fold increase in the risk of older people developing late‐onset familial and sporadic AD was caused by ApoE ε4 homozygotes compared to that due to other mutations (Rhinn et al., 2013). Previous studies have shown significant volume loss in the hippocampus and amygdala in homozygous ε4 carriers with AD compared with noncarriers (Filippini et al., 2009; Liu et al., 2010; Tanaka et al., 1998). However, few studies have been performed to evaluate the prediction of an ApoE ε4 carrier with images (Honea et al., 2009; Kim et al., 2013; Yim et al., 2022). The classification of AD from CN was investigated using a combination of fluorodeoxyglucose positron emission tomography, structural MRI, CSF protein levels, and apolipoprotein‐E (ApoE) genotype with an SVM ML method (Gupta et al., 2019). In addition, the contribution of ApoE 4 to modify the associations between GMV and cognitive impairment was investigated, suggesting a shift in the positive association between GMV and cognitive performance due to cholinergic neuronal dysfunction resulting from ApoE 4 status (Cacciaglia et al., 2019). The age at onset of AD and neuropathologic progression (Lahiri et al., 2004) and the rate of cognitive decline (Martins et al., 2005) were predicted by the ApoE genotype. Our finding is supported by results of previous studies that ApoE 4 existence can be predicted with GMV loss and cognitive impairment.

In addition, the results of the ROC curve analysis showed that the AUC value was .758 when both age and MMSE scores were combined as features (Table 2). Including GMVs in the amygdala, hippocampus, or MTG with the demographic values of both age and MMSE scores did not improve AUC value a lot (AUC = .747–.760), indicating that age and MMSE score are important predictors. The ML method is more helpful to evaluate predictions because multiple combinations can be analyzed faster and the high impact index can be picked up easier by it than ROC analysis.

Results of the three‐group classification with the different ML models showed that the largest AUC values were .830 for DIR GMV and .763 for T1W GMV (Table 5). Although AUC values were different among the ML models, the classification among the three participant groups was better DIR GMV than T1W GMV, indicating that GMV obtained with a DIR sequence can be used to differentiate the participant groups.

**TABLE 5:** Results of three‐group classification with the different machine learning (ML) models using both demographic data and gray matter volume (GMV) values obtained from six different brain areas for each imaging sequence.

ML models	T1W (AUC)	DIR (AUC)
**SVM1**	.763	.729
**SVM2**	.704	.752
**SVM3**	.695	.830
**Tree Bagger**	.727	.827
**Naive Bayes**	.761	.722

*Note*: The largest area‐under‐curve (AUC) values listed in this table were always obtained using 10‐feature combination, which are age, sex, mini–mental state examination (MMSE) scores, apolipoprotein E (ApoE) state, and 6 region‐of‐interest (ROI)s’ GMV values for each sequence. Therefore, we used all 10 features for both sequences. Features of the demographic data included in this analysis were age, sex, MMSE scores, and ApoE state. GMV values were used in the mean value if ROI had both the right and left sides. Machine learning (ML) models: support vector machine (SVM)1, the first‐order SVM or linear SVM model; SVM2, the second‐order polynomial SVM model or the quadratic SVM; SVM3, the third‐order polynomial SVM or cubic SVM or random forest model; TB, the Bootstrap‐Aggregated decision tree or Tree Bagger model; NB, the kernel‐based naive Bayes or kernel‐naive Bayes model.

Abbreviations: DIR, double inversion recover; T1W, T1‐weighted.

### 4.3 Study limitations

This study had some limitations. First, the number of participants was relatively small, especially the ApoE carrier group. Therefore, our results need to be validated by performing this study with a relatively large population. Second, all participants enrolled in our study were Korean. Due to the different effects of AopE ε4 status on different demographic distributions (Farrer et al., 1997), our study may be difficult to cover diverse cohorts. It is necessary to investigate the association between ApoE ε4 status and GMV in other demographic cohorts in future studies. Third, a prediction analysis was performed using the ML method with threefold cross‐validation. This validation can be increased up to 10 folds with increasing the number of participants. Finally, the MMSE score was used for cognitive function in this study. Other cognitive functions, such as attention, memory, language, visuospatial function, and frontal/executive function, may need to be investigated.

## 5 CONCLUSION

The combination of the five features of age, MMSE score, and DIR GMVs at the amygdala, hippocampus, and precuneus obtained the largest AUC value with the cubic SVM (SVM3) model. The carrier group showed significantly lower GMV than the noncarrier group at the bilateral amygdala, right precuneus, and right MTG. Our finding from the prediction analysis suggests that the ApoE ε4 genotype might be carried by an older participant with a low MMSE score and GMV reduction in the amygdala and hippocampus. This result is important to identify individuals who have a high risk for AD progression in the future.

## AUTHOR CONTRIBUTIONS

**Hyug‐Gi Kim**: Data curation; formal analysis; methodology; software; visualization; writing—original draft; writing—review and editing. **Yunan Tian**: Data curation; formal analysis; methodology; visualization; writing—original draft; writing—review and editing. **Sue Min Jung**: Data curation; formal analysis; methodology; visualization; writing—original draft; writing—review and editing. **Soonchan Park**: Resources; writing—original draft; writing—review and editing. **Hak Young Rhee**: Conceptualization; data curation; investigation; project administration; resources; supervision; writing—original draft; writing—review and editing. **Chang‐Woo Ryu**: Resources; writing—original draft; writing—review and editing. **Geon‐Ho Jahng**: Conceptualization; data curation; funding acquisition; investigation; methodology; project administration; software; supervision; validation; visualization; writing—original draft; writing—review and editing.

## CONFLICT OF INTEREST STATEMENT

We declare that we have no conflicts of interest.

### PEER REVIEW

The peer review history for this article is available at https://publons.com/publon/10.1002/brb3.3381.

## Supporting information

Supporting Information

## Data Availability

The raw data supporting the conclusions of this article will be made available by the corresponding author, without undue reservation.

## References

[brb33381-bib-0001] Ahn, H.‐J. , Chin, J. , Park, A. , Lee, B. H. , Suh, M. K. , Seo, S. W. , & Na, D. L. (2010). Seoul Neuropsychological Screening Battery‐dementia version (SNSB‐D): A useful tool for assessing and monitoring cognitive impairments in dementia patients. Journal of Korean Medical Science, 25, 1071–1076. 10.3346/jkms.2010.25.7.1071 20592901 PMC2890886

[brb33381-bib-0002] Ashburner, J. (2007). A fast diffeomorphic image registration algorithm. NeuroImage, 38, 95–113. 10.1016/j.neuroimage.2007.07.007 17761438

[brb33381-bib-0003] Bailey, H. R. , Sargent, J. Q. , Flores, S. , Nowotny, P. , Goate, A. , & Zacks, J. M. (2015). APOE epsilon4 genotype predicts memory for everyday activities. Neuropsychology, Development, and Cognition. Section B, Aging, Neuropsychology and Cognition, 22, 639–666. 10.1080/13825585.2015.1020916 PMC4537694 25754878

[brb33381-bib-0004] Breijyeh, Z. , & Karaman, R. (2020). Comprehensive review on Alzheimer's disease: Causes and treatment. Molecules (Basel, Switzerland), 25, 5789. 10.3390/molecules25245789 33302541 PMC7764106

[brb33381-bib-0005] Cacciaglia, R. , Molinuevo, J. L. , Falcón, C. , Sánchez‐Benavides, G. , Gramunt, N. , Brugulat‐Serrat, A. , Esteller, M. , Morán, S. , Fauria, K. , & Gispert, J. D. (2019). APOE‐epsilon4 risk variant for Alzheimer's disease modifies the association between cognitive performance and cerebral morphology in healthy middle‐aged individuals. NeuroImage: Clinical, 23, 101818. 10.1016/j.nicl.2019.101818 30991302 PMC6463204

[brb33381-bib-0006] Corder, E. H. , Saunders, A. M. , Strittmatter, W. J. , Schmechel, D. E. , Gaskell, P. C. , Small, G. W. , Roses, A. D. , Haines, J. L. , & Pericak‐Vance, M. A. (1993). Gene dose of apolipoprotein E type 4 allele and the risk of Alzheimer's disease in late onset families. Science, 261, 921–923. 10.1126/science.8346443 8346443

[brb33381-bib-0007] Coughlan, G. , Zhukovsky, P. , Voineskos, A. , & Grady, C. (2021). A profile of brain reserve in adults at genetic risk of Alzheimer's disease. Alzheimer's & Dementia (Amsterdam, Netherlands), 13, e12208. 10.1002/dad2.12208 PMC8190533 34136636

[brb33381-bib-0008] Diaz‐De‐Grenu, L. Z. , Acosta‐Cabronero, J. , Pereira, J. M. S. , Pengas, G. , Williams, G. B. , & Nestor, P. J. (2011). MRI detection of tissue pathology beyond atrophy in Alzheimer's disease: Introducing T2‐VBM. NeuroImage, 56, 1946–1953. 10.1016/j.neuroimage.2011.03.082 21473918

[brb33381-bib-0009] Dubois, B. , Feldman, H. H. , Jacova, C. , Dekosky, S. T. , Barberger‐Gateau, P. , Cummings, J. , Delacourte, A. , Galasko, D. , Gauthier, S. , Jicha, G. , Meguro, K. , O'brien, J. , Pasquier, F. , Robert, P. , Rossor, M. , Salloway, S. , Stern, Y. , Visser, P. J. , & Scheltens, P. (2007). Research criteria for the diagnosis of Alzheimer's disease: Revising the NINCDS‐ADRDA criteria. Lancet Neurology, 6, 734–746. 10.1016/S1474-4422(07)70178-3 17616482

[brb33381-bib-0010] Eichinger, P. , Kirschke, J. S. , Hoshi, M.‐M. , Zimmer, C. , Mühlau, M. , & Riederer, I. (2017). Pre‐ and postcontrast 3D double inversion recovery sequence in multiple sclerosis: A simple and effective MR imaging protocol. AJNR American Journal of Neuroradiology, 38, 1941–1945. 10.3174/ajnr.A5329 28751518 PMC7963634

[brb33381-bib-0011] Farrer, L. A. (1997). Effects of age, sex, and ethnicity on the association between apolipoprotein E genotype and Alzheimer disease. A meta‐analysis. APOE and Alzheimer Disease Meta Analysis Consortium. JAMA, 278, 1349–1356. 10.1001/jama.1997.03550160069041 9343467

[brb33381-bib-0012] Filippini, N. , Rao, A. , Wetten, S. , Gibson, R. A. , Borrie, M. , Guzman, D. , Kertesz, A. , Loy‐English, I. , Williams, J. , Nichols, T. , Whitcher, B. , & Matthews, P. M. (2009). Anatomically‐distinct genetic associations of APOE epsilon4 allele load with regional cortical atrophy in Alzheimer's disease. NeuroImage, 44, 724–728. 10.1016/j.neuroimage.2008.10.003 19013250

[brb33381-bib-0013] Geurts, J. J. G. , Pouwels, P. J. W. , Uitdehaag, B. M. J. , Polman, C. H. , Barkhof, F. , & Castelijns, J. A. (2005). Intracortical lesions in multiple sclerosis: Improved detection with 3D double inversion‐recovery MR imaging. Radiology, 236, 254–260. 10.1148/radiol.2361040450 15987979

[brb33381-bib-0014] Gupta, Y. , Lama, R. K. , & Kwon, G.‐R. , Alzheimer's Disease Neuroimaging Initiative . (2019). Prediction and classification of Alzheimer's disease based on combined features from apolipoprotein‐E genotype, cerebrospinal fluid, MR, and FDG‐PET imaging biomarkers. Frontiers in Computational Neuroscience, 13, 72. 10.3389/fncom.2019.00072 31680923 PMC6805777

[brb33381-bib-0015] Hashimoto, M. , Yasuda, M. , Tanimukai, S. , Matsui, M. , Hirono, N. , Kazui, H. , & Mori, E. (2001). Apolipoprotein E epsilon 4 and the pattern of regional brain atrophy in Alzheimer's disease. Neurology, 57, 1461–1466. 10.1212/WNL.57.8.1461 11673590

[brb33381-bib-0016] Honea, R. A. , Vidoni, E. , Harsha, A. , & Burns, J. M. (2009). Impact of APOE on the healthy aging brain: A voxel‐based MRI and DTI study. Journal of Alzheimer's Disease, 18, 553–564. 10.3233/JAD-2009-1163 PMC2892293 19584447

[brb33381-bib-0017] Jahng, G.‐H. , Lee, D. K. , Lee, J.‐M. , Rhee, H. Y. , & Ryu, C.‐W. (2016). Double inversion recovery imaging improves the evaluation of gray matter volume losses in patients with Alzheimer's disease and mild cognitive impairment. Brain Imaging and Behavior, 10, 1015–1028. 10.1007/s11682-015-9469-2 26497891

[brb33381-bib-0018] Kim, H.‐G. , Park, S. , Rhee, H. Y. , Lee, K. M. , Ryu, C.‐W. , Lee, S. Y. , Kim, E. J. , Wang, Y. , & Jahng, G.‐H. (2020). Evaluation and prediction of early Alzheimer's disease using a machine learning‐based optimized combination‐feature set on gray matter volume and quantitative susceptibility mapping. Current Alzheimer Research, 17, 428–437. 10.2174/1567205017666200624204427 32579502

[brb33381-bib-0019] Kim, S. M. , Kim, M. J. , Rhee, H. Y. , Ryu, C.‐W. , Kim, E. J. , Petersen, E. T. , & Jahng, G.‐H. (2013). Regional cerebral perfusion in patients with Alzheimer's disease and mild cognitive impairment: Effect of APOE epsilon4 allele. Neuroradiology, 55, 25–34. 10.1007/s00234-012-1077-x 22828738

[brb33381-bib-0020] Lahiri, D. K. , Sambamurti, K. , & Bennett, D. A. (2004). Apolipoprotein gene and its interaction with the environmentally driven risk factors: Molecular, genetic and epidemiological studies of Alzheimer's disease. Neurobiology of Aging, 25, 651–660. 10.1016/j.neurobiolaging.2003.12.024 15172744

[brb33381-bib-0021] Liu, Y. , Paajanen, T. , Westman, E. , Wahlund, L.‐O. , Simmons, A. , Tunnard, C. , Sobow, T. , Proitsi, P. , Powell, J. , Mecocci, P. , Tsolaki, M. , Vellas, B. , Muehlboeck, S. , Evans, A. , Spenger, C. , Lovestone, S. , & Soininen, H. , The AddNeuroMed Consortium . (2010). Effect of APOE epsilon4 allele on cortical thicknesses and volumes: The AddNeuroMed study. Journal of Alzheimer's Disease, 21, 947–966. 10.3233/JAD-2010-100201 20693633

[brb33381-bib-0022] Lumsden, A. L. , Mulugeta, A. , Zhou, A. , & Hyppönen, E. (2020). Apolipoprotein E (APOE) genotype‐associated disease risks: A phenome‐wide, registry‐based, case‐control study utilising the UK Biobank. EBioMedicine, 59, 1–11. 10.1016/j.ebiom.2020.102954 PMC7452404 32818802

[brb33381-bib-0023] Manning, C. D. , Raghavan, P. , & Schütze, H. (2008). Introduction to information retrieval. Cambridge University Press.

[brb33381-bib-0024] Martins, C. A. R. , Oulhaj, A. , De Jager, C. A. , & Williams, J. H. (2005). APOE alleles predict the rate of cognitive decline in Alzheimer disease: A nonlinear model. Neurology, 65, 1888–1893. 10.1212/01.wnl.0000188871.74093.12 16380608

[brb33381-bib-0025] Mckhann, G. , Drachman, D. , Folstein, M. , Katzman, R. , Price, D. , & Stadlan, E. M. (1984). Clinical diagnosis of Alzheimer's disease: Report of the NINCDS‐ADRDA Work Group under the auspices of Department of Health and Human Services Task Force on Alzheimer's Disease. Neurology, 34, 939–944. 10.1212/WNL.34.7.939 6610841

[brb33381-bib-0026] Meinshausen, N. (2006). Quantile regression forests. Journal of Machine Learning Research, 7, 983–999.

[brb33381-bib-0027] Molinder, A. , Ziegelitz, D. , Maier, S. E. , & Eckerström, C. (2021). Validity and reliability of the medial temporal lobe atrophy scale in a memory clinic population. BMC Neurology, [Electronic Resource], 21, 289. 10.1186/s12883-021-02325-2 34301202 PMC8305846

[brb33381-bib-0028] Nicoll, J. A. R. , Savva, G. M. , Stewart, J. , Matthews, F. E. , Brayne, C. , & Ince, P. (2011). Association between APOE genotype, neuropathology and dementia in the older population of England and Wales. Neuropathology and Applied Neurobiology, 37, 285–294. 10.1111/j.1365-2990.2010.01130.x 20880354

[brb33381-bib-0029] Petersen, R. C. (2016). Mild cognitive impairment. Continuum (Minneap Minn), 22, 404–418. 27042901 10.1212/CON.0000000000000313 PMC5390929

[brb33381-bib-0030] Petersen, R. C. , Doody, R. , Kurz, A. , Mohs, R. C. , Morris, J. C. , Rabins, P. V. , Ritchie, K. , Rossor, M. , Thal, L. , & Winblad, B. (2001). Current concepts in mild cognitive impairment. Archives of Neurology, 58, 1985–1992. 11735772 10.1001/archneur.58.12.1985

[brb33381-bib-0031] Petersen, R. C. , Smith, G. E. , Waring, S. C. , Ivnik, R. J. , Tangalos, E. G. , & Kokmen, E. (1999). Mild cognitive impairment: Clinical characterization and outcome. Archives of Neurology, 56, 303–308. 10.1001/archneur.56.3.303 10190820

[brb33381-bib-0032] Pini, L. , Pievani, M. , Bocchetta, M. , Altomare, D. , Bosco, P. , Cavedo, E. , Galluzzi, S. , Marizzoni, M. , & Frisoni, G. B. (2016). Brain atrophy in Alzheimer's disease and aging. Ageing Research Reviews, 30, 25–48. 10.1016/j.arr.2016.01.002 26827786

[brb33381-bib-0033] Pouwels, P. J. W. , Kuijer, J. P. A. , Mugler, J. P. , Guttmann, C. R. G. , & Barkhof, F. (2006). Human gray matter: Feasibility of single‐slab 3D double inversion‐recovery high‐spatial‐resolution MR imaging. Radiology, 241, 873–879. 10.1148/radiol.2413051182 17053197

[brb33381-bib-0034] Rhinn, H. , Fujita, R. , Qiang, L. , Cheng, R. , Lee, J. H. , & Abeliovich, A. (2013). Integrative genomics identifies APOE epsilon4 effectors in Alzheimer's disease. Nature, 500, 45–50. 10.1038/nature12415 23883936

[brb33381-bib-0035] Suzuki, K. , Hirakawa, A. , Ihara, R. , Iwata, A. , Ishii, K. , Ikeuchi, T. , Sun, C.‐K. , Donohue, M. , & Iwatsubo, T. , Alzheimer's Disease Neuroimaging Initiative, & Japanese Alzheimer's Disease Neuroimaging Initiative . (2020). Effect of apolipoprotein E epsilon4 allele on the progression of cognitive decline in the early stage of Alzheimer's disease. Alzheimer's and Dementia (New York, N. Y.), 6, e12007. 10.1002/trc2.12007 PMC7087431 32211510

[brb33381-bib-0036] Tanaka, S. , Kawamata, J. , Shimohama, S. , Akaki, H. , Akiguchi, I. , Kimura, J. , & Ueda, K. (1998). Inferior temporal lobe atrophy and APOE genotypes in Alzheimer's disease. X‐ray computed tomography, magnetic resonance imaging and Xe‐133 SPECT studies. Dementia and Geriatric Cognitive Disorders, 9, 90–98. 10.1159/000017029 9524800

[brb33381-bib-0037] Vemuri, P. , Wiste, H. J. , Weigand, S. D. , Knopman, D. S. , Shaw, L. M. , Trojanowski, J. Q. , Aisen, P. S. , Weiner, M. , Petersen, R. C. , & Jack, C. R., Jr. , Alzheimer's Disease Neuroimaging Initiative . (2010). Effect of apolipoprotein E on biomarkers of amyloid load and neuronal pathology in Alzheimer disease. Annals of Neurology, 67, 308–316. 10.1002/ana.21953 20373342 PMC2886799

[brb33381-bib-0038] Verghese, P. B. , Castellano, J. M. , & Holtzman, D. M. (2011). Apolipoprotein E in Alzheimer's disease and other neurological disorders. Lancet Neurology, 10, 241–252. 10.1016/S1474-4422(10)70325-2 21349439 PMC3132088

[brb33381-bib-0039] Yim, Y. , Choi, J. D. , Cho, J. H. , Moon, Y. , Han, S.‐H. , & Moon, W.‐J. (2022). Magnetic susceptibility in the deep gray matter may be modulated by apolipoprotein E4 and age with regional predilections: A quantitative susceptibility mapping study. Neuroradiology, 64, 1331–1342. 10.1007/s00234-021-02859-9 34981175

