# Supplementary material for: Predicting the apolipoprotein E ε4 allele carrier status based on gray matter volumes and cognitive function
Source: Brain Behav. 2024 Jan 8;14(1):e3381. doi: 10.1002/brb3.3381 (PMC10772845; doi:10.1002/brb3.3381)
Supplement: Supplementary file 1 — Supporting Information [file BRB3-14-e3381-s001.docx]

**Predicting the apolipoprotein E ε4 allele carrier status based on gray matter volumes and cognitive function**

**Supplementary Results**

**Supplementary Table S1.** Areas with significant differences of the gray matter volume (GMV) between apolipoprotein E (ApoE) ε4 allele carriers (C) and non-carriers (NC) in the voxel-based comparison using a two-sample t test for the three-dimensional T1-weighted (T1W) and double inversion recovery (DIR) sequence.

| **Group analysis** | **Cluster size** | **Cluster location** | **BA** | **Talairach coordinates** | **Z score** |
| --- | --- | --- | --- | --- | --- |
| **T1W GMV** | | | | | |
| NC>C | 3602 | Lt Parietal Precuneus | 7 | -1.66, -33.9, 44.28 | 5.23 |
|  |  | Rt Occipital Cuneus | 19 | 11.23, -78.33, 36.68 | 4.11 |
|  |  | Lt Parietal Precuneus | 7 | -7.37, -51.51, 51.52 | 4.00 |
|  | 8162 | Rt Medial Frontal Gyrus | 11 | 8.6, 33.81, -13.99 | 4.89 |
|  |  | Rt Superior Temporal Gyrus | 38 | 34.58, 14.05, -24.44 | 4.76 |
|  |  | Rt Limbic Cingulate Gyrus | 32 | 2.29, 19.13, 40.36 | 4.38 |
|  | 1452 | Lt Parietal Postcentral Gyrus | 3 | -24.17, -32.32, 67.47 | 4.74 |
|  |  | Lt Frontal Precentral Gyrus | 4 | -49.65, -8.9, 40.43 | 4.03 |
|  | 875 | Rt Occipital Fusiform Gyrus | 19 | 39.62, -77.5, -11.4 | 4.50 |
|  |  | Rt Middle Temporal Gyrus | 37 | 44.93, -70.13, 9.2 | 3.93 |
|  |  | Rt Middle Occipital Gyrus | 18 | 22.68, -86.78, 7.25 | 3.68 |
|  | 1433 | Lt Temporal Subcallosal Gyrus | 34 | -28.56, 5.53, -11.9 | 4.24 |
|  |  | Lt Lentiform Nucleus, Putamen |  | -21.23, 21.39, -1.26 | 3.90 |
|  |  | Lt Superior Temporal Gyrus | 38 | -39.56, 10.02, -18.87 | 3.47 |
|  | 243 | Lt Inferior Occipital Gyrus | 19 | -40.14, -80.17, -0.39 | 3.95 |
|  | 312 | Rt Middle Temporal Gyrus | 21 | 56.4, -20, -3.87 | 3.70 |
|  |  | Rt Superior Temporal Gyrus | 22 | 43.5, -25, -9.97 | 3.223 |
|  | 100 | Rt Inferior Temporal Gyrus | 20 | 36.54, -7.09, -39.02 | 3.66 |
|  | 149 | Lt Middle Temporal Gyrus | 21 | -56.78, -56.21, 5.2 | 3.65 |
|  | 108 | Lt Frontal Precentral Gyrus | 6 | -32.9, -1.01, 36.06 | 3.50 |
|  | 177 | Rt Frontal Precentral Gyrus | 6 | 43.04, 0.81, 33.91 | 3.43 |
|  |  | Rt Middle Frontal Gyrus | 6 | 41.05, 1.63, 44.76 | 3.41 |
|  | 284 | Lt Middle Occipital Gyrus | 19 | -40.3, -79.52, 12.28 | 3.38 |
|  |  | Lt Superior Occipital Gyrus | 19 | -33.09, -82.82, 26.5 | 3.16 |
|  | 140 | Rt Frontal Precentral Gyrus | 4 | 11.07, -30.12, 62.87 | 3.36 |
|  |  | Rt Parietal Postcentral Gyrus | 3 | 14.7, -34.38, 67.93 | 3.30 |
| NC<C | none |  |  |  |  |
| **DIR GMV** | | | | | |
| NC>C | 884 | Rt Middle Frontal Gyrus | 46 | 50.65, 20.62, 23.3 | 5.35 |
|  |  | Rt Inferior Frontal Gyrus | 10 | 39.97, 49.37, -1.18 | 4.67 |
|  | 737 | Lt Parietal Precuneus | 31 | -8.95, -69.55, 24.56 | 4.96 |
|  |  | Lt Limbic Posterior Cingulate | 30 | -6.88, -58.85, 11.2 | 4.62 |
|  |  | Rt Limbic Posterior Cingulate | 30 | 13.43, -57.44, 15.28 | 4.49 |
|  | 419 | Lt Limbic Uncus | 36 | -26.51, -9.5, -31.3 | 4.89 |
|  |  | Lt Lentiform Nucleus, Putamen |  | -17.45, 7.34, -11.54 | 4.45 |
|  | 514 | Lt Medial Frontal Gyrus | 10 | -8.07, 63.01, -4.3 | 4.86 |
|  |  | Lt Superior Frontal Gyrus | 10 | -19.22, 55.44, -3.41 | 4.39 |
|  | 361 | Rt Middle Temporal Gyrus | 22 | 50.64, -36.1, 7.12 | 4.68 |
|  |  | Rt Superior Temporal Gyrus | 21 | 58.25, -38.29. -9.18 | 4.02 |
|  | 448 | Rt Superior Frontal Gyrus | 10 | 14.15, 57.48, -6.25 | 4.60 |
|  |  | Rt Middle Frontal Gyrus | 10 | 23.27, 61.98, 5.14 | 4.42 |
|  |  | Rt Medial Frontal Gyrus | 10 | 14.04, 60.34, 3.02 | 4.03 |
|  | 188 | Lt Frontal Paracentral Lobule | 31 | -5.27, -9.48, 44.73 | 4.55 |
|  |  | Lt Limbic Cingulate Gyrus | 31 | -10.84, -22.32, 41.62 | 3.90 |
|  | 256 | Lt Inferior Frontal Gyrus | 47 | -41.55, 25.4, -3.03 | 4.54 |
|  | 197 | Lt Frontal Precentral Gyrus | 6 | -51.09, 16.08, 15.75 | 4.47 |
|  | 340 | Rt Sub-Lobar Claustrum | * | 37.9, 0.24, 1.34 | 4.43 |
|  | 165 | Rt Frontal Precentral Gyrus | 4 | 57.85, -13.83, 29.17 | 4.38 |
|  | 427 | Rt Limbic Uncus | 28 | 23.44, -9.94, -28.7 | 4.33 |
|  |  | Rt Limbic Anterior Cingulate | 25 | 6.6, 12.63, -8.83 | 4.06 |
|  |  | Rt Limbic Parahippocampal Gyrus | 28 | 17.77, -10.78, -19.87 | 3.92 |
|  | 175 | Lt Occipital Lingual Gyrus | 18 | -10.52, -78.46, 0.27 | 4.27 |
|  | 289 | Lt Parietal Precuneus | 7 | -7.36, -56.92, 49.21 | 3.97 |
|  |  | Lt Parietal Precuneus | 7 | -3.05, -55.69, 36.68 | 3.88 |
|  |  | Rt Parietal Precuneus | 7 | 1.87, -64.42, 48.65 | 3.82 |
| NC<C | none |  |  |  |  |

The significance level was set at α = 0.05 with correcting multiple comparisons using the false discovery rate (FDR) method and with the minimum cluster size with at least 100 contiguous voxels.

In this table, we list the cluster locations with Z-score more than 3.0 and with the cluster size more than 100.

The similar result is graphically shown in **Figure 1**.

Abbreviations: BA, Brodmann area; Lt, left; Rt, right;

**Supplementary Table S2.** Result of the two-sample t-test analysis of the gray matter volume (GMV) between apolipoprotein E (ApoE) ε4 carrier (C) and non-carrier (NC) groups in the specific region-of-interest (ROI) areas for T1-weighted (T1W) and double inversion recovery (DIR) image

| **ROIs** | **Sequence** | | **Subject groups** | | **Statistical result**  **p-value** |
| --- | --- | --- | --- | --- | --- |
|  |  |  | **Noncarrier (NC)** | **Carrier (C)** |  |
| Amygdala | T1W | Lt | 0.550±0.003 | 0.514±0.003 | *0.015* |
|  |  | Rt | 0.496±0.002 | 0.465±0.002 | *0.009* |
|  | DIR | Lt | 0.748±0.001 | 0.719±0.002 | *0.003* |
|  |  | Rt | 0.691±0.001 | 0.671±0.001 | *0.006* |
| Hippocampus | T1W | Lt | 0.446±0.002 | 0.437±0.002 | 0.448 |
|  |  | Rt | 0.413±0.002 | 0.404±0.002 | 0.378 |
|  | DIR | Lt | 0.559±0.002 | 0.536±0.002 | *0.026* |
|  |  | Rt | 0.523±0.002 | 0.495±0.001 | *0.013* |
| Precuneus | T1W | Lt | 0.366±0.001 | 0.349±0.002 | 0.055 |
|  |  | Rt | 0.379±0.001 | 0.354±0.001 | *0.004* |
|  | DIR | Lt | 0.547±0.001 | 0.519±0.002 | *0.001* |
|  |  | Rt | 0.570±0.0004 | 0.545±0.002 | *0.002* |
| PC | T1W | Lt | 0.327±0.001 | 0.310±0.003 | 0.107 |
|  |  | Rt | 0.256±0.001 | 0.245±0.001 | 0.178 |
|  | DIR | Lt | 0.458±0.0003 | 0.451±0.001 | 0.140 |
|  |  | Rt | 0.347±0.001 | 0.343±0.001 | 0.505 |
| MFG | T1W | Lt | 0.348±0.002 | 0.335±0.001 | 0.167 |
|  |  | Rt | 0.355±0.001 | 0.340±0.001 | 0.094 |
|  | DIR | Lt | 0.483±0.001 | 0.467±0.001 | *0.025* |
|  |  | Rt | 0.504±0.001 | 0.489±0.001 | *0.023* |
| MTG | T1W | Lt | 0.413±0.002 | 0.400±0.001 | 0.128 |
|  |  | Rt | 0.413±0.002 | 0.386±0.002 | *0.015* |
|  | DIR | Lt | 0.594±0.001 | 0.581±0.001 | *0.046* |
|  |  | Rt | 0.578±0.0004 | 0.568±0.0004 | *0.050* |

Data list mean ± standard deviation (SD). The p-value indicates the result of the two-sample t-test of gray matter volume between ApoE ε4 allele carriers and non-carriers.

The similar result is graphically shown in **Figure 2**.

Abbreviations: Lt, left; Rt, right; PC, Posterior Cingulate; MFG, Middle Frontal Gyrus; MTG, Middle Temporal Gyrus


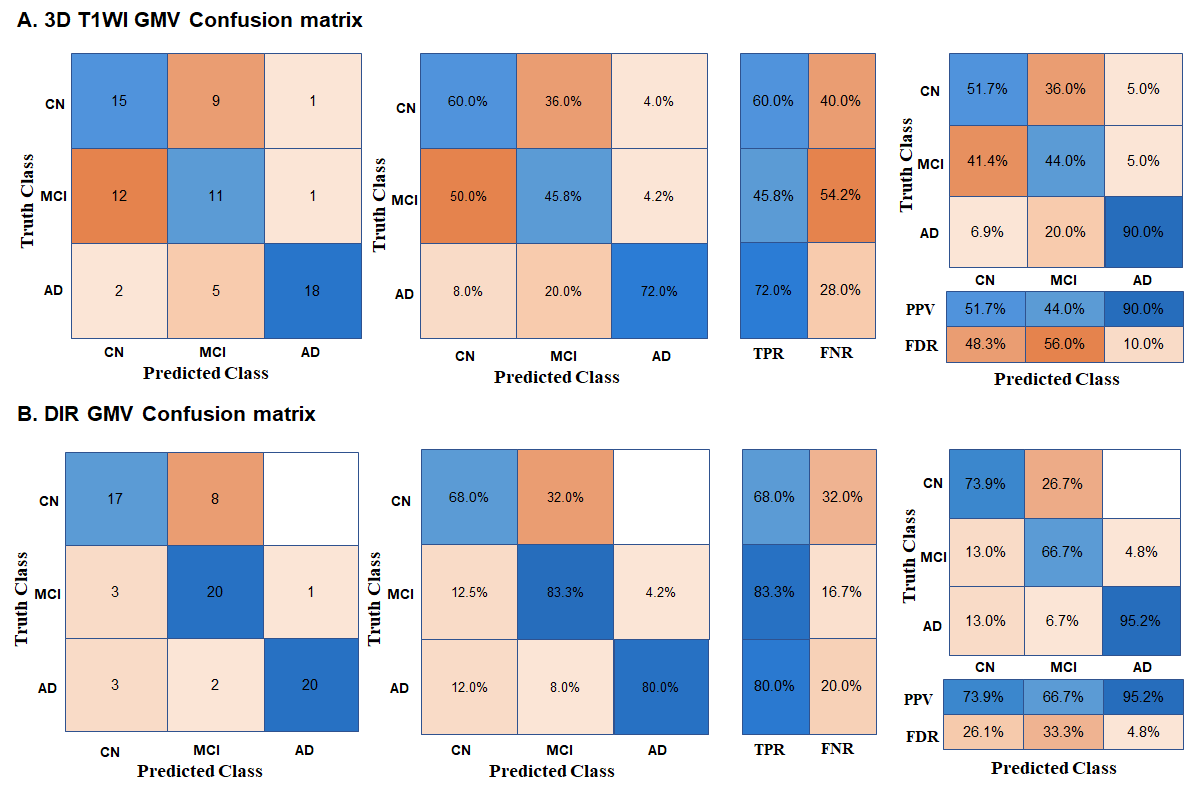


**Supplementary Figure S1.** Confusion matrices of the 3rd order polynomial support vector machine (SVM) or cubic SVM (SVM3) for the classification among the three participant groups with the gray matter volume (GMV) obtained with the three-dimensional (3D) T1-weighted image (T1W) (A) or double inversion recovery (DIR) (B) sequence.

Each row corresponds to the true grade and column corresponds to the predicted grade. The blue color at the main diagonal represents the number of correctly classified data points. Numbers off the diagonal represent the number of misclassified data points.

Abbreviations: CN, cognitively normal; MCI, mild cognitive impairment; AD, Alzheimer’s disease; TPR, true positive rate; FNR, false negative rate; PPV, positive predictive value; FDR, false discovery rate.
